# Clinical outcomes from blended care therapy for anxiety and depression in the year after treatment

**DOI:** 10.1016/j.invent.2024.100798

**Published:** 2024-12-28

**Authors:** Jennifer L. Lee, Shih-Yin Chen, Robert E. Wickham, Pam Wang, Monica S. Wu, Alethea A. Varra, Connie E. Chen, Anita Lungu

**Affiliations:** a Lyra Health, 270 East Lane Burlingame, CA 94010, United States of America; b Emory University, 1405 Clifton Rd. NE, Atlanta, GA 30307, United States of America; c Department of Psychological Sciences, Northern Arizona University, 1100 S Beaver St, Student Academic Services, Flagstaff, AZ 86011, United States of America

**Keywords:** Blended care therapy, Mental health, Anxiety, Depression, Longitudinal outcomes, 12-month follow-up

## Abstract

**Background:**

Scalable evidence-based treatments for anxiety and depression, such as blended care therapy (BCT) that integrate digital tools are effective, but reporting on long-term outcomes is limited.

**Method:**

This pragmatic observational study examined the symptom stability and trajectories of individuals in the year following engagement in a BCT program. Participants included adults with clinical anxiety and/or depression measured by the Generalized Anxiety Disorder-7 (GAD-7) or Patient Health Questionnaire-9 (PHQ-9). Assessments were sent during the initial episode of care and in the year following.

**Results:**

Participants included 27,835 adults (depression: 17,686 and anxiety: 24,853). Of these, 11,465 (41 % of those who received initial care; depression: 7223; anxiety: 10,218) completed at least one follow-up assessment (FUA). Average age was 34 years, 68–69 % were female, and 48–49 % were White across subsamples. Among FUA respondents, rates of reliable improvement or recovery on the PHQ-9 or GAD-7 for those who did not receive additional therapy were above 81 % across follow-up periods. Growth curve analysis for those who did not return for additional therapy revealed that both depression and anxiety groups demonstrated a statistically significant yet small linear effect of time in the year following treatment, with a 1.6–2.1 point increase in scores over the 12-month period.

**Conclusions:**

Among clients who completed FUAs and received no additional therapy, reliable improvement and recovery rates were high. Growth curve analysis demonstrated a small increase in symptoms over the 12-month interval, providing pragmatic evidence of long-term stability of treatment gains from BCT for anxiety and depression in a real-world setting.

## Highlights

• Blended care therapy (BCT) can be a scalable solution for addressing anxiety and depression
• Little is known about the long-term outcomes from BCT in the year following treatment
• For those who received no additional therapy, reported symptom improvements in anxiety and depression were stable and high
• A comparatively small proportion of individuals returned for additional therapy
• Additional long-term pragmatic research is needed, given challenges of obtaining naturalistic follow-up data

## 1 Introduction

Mental health challenges are common, with the prevalence of mental health disorders nearly doubling over the last 30 years (GBD, 2016 Disease and Injury Incidence and Prevalence Collaborators, 2017; Teixeira et al., 2022). Symptoms of anxiety and depression are most common and result in substantial reductions in quality of life, billions of dollars in disability, and functional impairment (GBD, 2019 Mental Disorders Collaborators, 2022; Roehrig, 2016). Impairments in functioning due to symptoms of anxiety and depression occur across multiple life domains, often affecting home and work environments (GBD, 2019 Mental Disorders Collaborators, 2022; Grasso et al., 2022). At home, depression and anxiety are related to difficulties in romantic and social relationships (Leach and Butterworth, 2020; Werner-Seidler et al., 2017). Having a mental health disorder is also associated with greater likelihood of working while impaired and missing work, resulting in significant lost wages (de Oliveira et al., 2023; Evans-Lacko and Knapp, 2016). Level of symptom severity on measures of depression and anxiety are also strongly correlated with functional impacts, including overall health functioning, healthcare utilization, and sick-days from work (Huang et al., 2006; Kroenke et al., 2001; Ruiz et al., 2011). While symptoms of depression and anxiety may fluctuate over time, clinical levels of anxiety and depression are often persistent and chronic. In a landmark study following individuals with depression and/or anxiety for 9 years, 79 % of individuals in the study experienced recurrence of their diagnosis (Solis et al., 2021).

Given the functional impact of these disorders and their chronicity, access to consistent and effective mental health care is critical. Unfortunately, accessing effective and appropriate evidence-based care is fraught with barriers (Andrade et al., 2014), including logistical barriers, such as physical distance from a therapist and state-based licensure rules, as well as knowledge barriers, such as not knowing whether a provider focuses on evidence-based care. Many of these barriers have been exacerbated by shortages in trained mental health providers that are anticipated to continue in the coming decades (National Center for Health Workforce Analysis, 2022). Blended care therapy (BCT) is a specific treatment modality that involves synchronous therapy sessions and asynchronous use of evidence-based digital clinical tools. This model of care maximizes the effects of treatment with reinforcement and support from therapists in synchronous therapy sessions. In the model investigated in this manuscript, all synchronous care is also delivered via telemental health using video, reducing logistical barriers. Meta-analyses have supported the effectiveness of blended care therapies for symptoms of anxiety and depression (Karyotaki et al., 2018; Newby et al., 2016; Păsărelu et al., 2017). Real-world evaluation of BCT demonstrated that it effectively decreases symptoms of anxiety and depression over the course of treatment, supported by high rates of reliable improvement, as well as clinical recovery (Lungu et al., 2022; Owusu et al., 2022; Wu et al., 2021). While these previous investigations speak to the effectiveness of BCT for improving symptoms of anxiety and depression while in care, long-term follow-up is needed to determine if these gains are maintained over time.

Randomized controlled trials (RCTs) for depression and anxiety using evidence-based treatments show high rates of relapse and recurrence in the year following treatment, with rates ranging from 34 % to 50 % in depression (Chen et al., 2022; Wojnarowski et al., 2019) and 13 % to 42 % in anxiety (Lorimer et al., 2021), highlighting the importance of long-term follow-up. One RCT has demonstrated stable depressive symptom improvement over a 1-year period for BCT, with 50–64 % of the sample demonstrating stable improved symptoms (Mathiasen et al., 2022, Mathiasen, Andersen, Riper, Kleiboer and Roessler, 2016). This study however included small sample sizes and was executed in a highly controlled research setting that is not reflective of everyday practice in the real world, where both outcomes and clinical quality are more variable (Kennedy-Martin et al., 2015).

Given the chronic nature of anxiety and depression, it is also anticipated that many individuals may return to care in the year following treatment. One of the few studies reporting on the rates of individuals who return to seek additional care in the year following treatment in Norway estimates these rates to fall between 25 % and 50 %, however these findings may be specific to the Norwegian healthcare system (Sæther et al., 2019). In the only identified study in the US from the Veterans Affairs Healthcare System, nearly 77 % of individuals who completed treatment returned for additional individual psychotherapy in the year following (Baier et al., 2024). Applied research in this area outside of national healthcare systems is extremely limited however, particularly in the United States, due to the challenges of collecting outcomes over extended periods of time outside of a formal research setting. Missing data are also a significant challenge to longitudinal research, and levels of missing data from patient-reported outcomes are often under-reported, even in clinical trials (Calvert et al., 2018). Missing data are a particular challenge when outcomes are collected as a part of real-world data in applied clinical practice, where no compensation is provided for assessment completion (Kendrick et al., 2016).

There is no known published research on the long-term stability of anxiety and depression symptoms in real-world settings for blended care therapy. This data is critical for demonstrating the long-term effectiveness of this treatment modality for anxiety and depression, given the high rates of relapse and recurrence for these diagnoses. Symptom monitoring following completion of care also helps to ensure the quality of treatment being received, particularly given ongoing concerns about the quality of mental health care delivered outside of clinical trials (Kilbourne et al., 2018). Despite repeated calls and recommendations for ongoing mental health monitoring at a population level (US Preventive Services Task Force, 2023a, US Preventive Services Task Force, 2023b), implementation of this recommendation is extremely limited and unsystematic. Follow-up treatment for those who do screen positive for concerns during monitoring is subject to the same substantial barriers encountered during initial treatment seeking, and could prevent timely re-engagement in care. However, data on the number of individuals who engage in care following routine monitoring are also limited.

This investigation represents a pragmatic longitudinal observation of individuals with clinical levels of anxiety and/or depressive symptoms at the start of care who engaged in a BCT program. Follow-up data was collected as a component of routine symptom and quality monitoring, including multiple assessment points during initial care and in the 1 year following treatment engagement. The primary objective is to evaluate the longitudinal stability of real-world clinical symptoms of anxiety and depression following engagement with a BCT program. The secondary objective of the study is to examine rates of re-engagement with therapy services in the year following initial treatment, including an exploratory analysis examining differences in symptoms and trajectories by re-engagement behavior. The study aims to fill a substantial gap in the literature, answering whether this type of therapy program generates improvements in clinical symptoms that are stable over time using a naturalistic study design evaluating everyday care delivery.

## 2 Methods

### 2.1 Study design

The investigation consisted of a real-world observational cohort design. Data were collected as a part of routine quality control and measurement-based care during a BCT program described below and in previous publications (Lungu et al., 2020). Treatment was provided as a component of an employer-sponsored workforce mental health program (WMHP). Informed consent for treatment was obtained from all participants. This retrospective analysis was completed on deidentified data collected as routine quality control for treatment offered by Lyra Clinical Associates, and was determined to be exempt research by the Western-Copernicus Group (WCG) Institutional Review Board.

### 2.2 Setting

Participants included adults in the United States who were eligible for Lyra Health's WMHP initiating care between September 2019 and February 2023, and have either completed or dropped out of care. Participants included employees or dependents from 168 employers, across all 50 states, the District of Columbia, and 4 United States territories.

### 2.3 Treatment description

BCT was provided via synchronous video therapy, integrating asynchronous digital care tools. Treatment was delivered via a secure, proprietary platform following procedures described in detail elsewhere (Lungu et al., 2022, Lungu, Jun, Azarmanesh, Leykin and Chen, 2020; Owusu et al., 2022). Individuals were able to re-engage in care at any point following the completion of an initial episode of BCT and were eligible for booster sessions (i.e., 1–2 sessions to refresh skills practice) as a part of routine care. They were also eligible to receive additional individual psychotherapy through WMHP's network of specialized providers or additional courses of BCT.

## 3 Measures

### 3.1 Demographics

Participants self-reported on their age, gender, and race and/or ethnicity through the registration and intake process.

### 3.2 Anxiety and depression symptoms

The Patient Health Questionnaire-9 (PHQ-9) (Kroenke et al., 2001) and Generalized Anxiety Disorder-7 (GAD-7) (Spitzer et al., 2006) were sent to participants as weekly assessments for depression and anxiety symptoms. Clinical cut-offs of PHQ-9 ≥ 10 and GAD-7 ≥ 8 were utilized for the baseline scores to determine inclusion in the sample. Individuals scoring above those thresholds would likely meet diagnostic criteria for major depression (Kroenke et al., 2001) or an anxiety disorder (Kroenke et al., 2007; Plummer et al., 2016). Rates of reliable improvement and recovery were calculated. Reliable improvement is determined by a psychometrically reliable decrease in total symptom score of 6 or more points on the PHQ-9, or a decrease in total symptom score of 4 or more points on the GAD-7 (Liness et al., 2019; Toussaint et al., 2020). Recovery is achieved by a total score on the PHQ-9 or GAD-7 falling in the subclinical range, which is <10 and <8, respectively. Specifically for follow-up assessments (FUA), the following classifications were used: Reliable Improvement: ≥ 4 decrease on the FUA GAD-7 among those with baseline GAD-7 ≥ 8 and/or ≥ 6 decrease on the FUA PHQ-9 among those with baseline PHQ-9 ≥ 10; Recovery: FUA GAD-7 < 8 among those with baseline GAD-7 ≥ 8 and/or FUA PHQ-9 < 10 among those with baseline PHQ-9 ≥ 10; Reliable Improvement and Recovery: ≥ 4 decrease on the FUA GAD-7 and FUA GAD-7 < 8 among those with baseline GAD-7 ≥ 8; and/or, ≥ 6 decrease on the FUA PHQ-9 and FUA PHQ-9 < 10 among those with baseline PHQ-9 ≥ 10; Reliable Improvement or Recovery: ≥ 4 decrease on the FUA GAD-7 or FUA GAD-7 < 8 among those with baseline GAD-7 ≥ 8; and/or, ≥ 6 decrease on the FUA PHQ-9 or FUA PHQ-9 < 10 among those with baseline PHQ-9 ≥ 10.

Follow-up assessments were sent to all individuals at 3, 6, 9, and 12 months following the end of treatment. As relapse and recurrence of symptoms is common with anxiety and depression, FUAs were intended to serve as intentional check-ins to monitor ongoing symptoms and provide feedback to clients on their total scores once assessments were completed. Participants were encouraged to re-engage in care and seek support if they felt it was needed, or if they reported suicidal ideation. Resources were provided, such as encouragement to contact their previous therapist, crisis service contact information, and contact information for WMHP care navigators to assist in being connected with mental health services. Individuals were classified for subgroup analyses based upon their post-care treatment engagement behavior using the following categories: those who received additional individual psychotherapy (AT), those engaging in additional booster sessions (AB) after their first episode of BCT, or those who received no additional individual psychotherapy (NAT). Individuals who engaged in additional care following their initial treatment episode were also sent routine in-care assessments for their second episode of care (i.e., PHQ-9 and/or GAD-7). See the Supplementary Information for additional details on assessments.

### 3.3 Patient population

Participants included individuals 18 years of age and older who enrolled in their first ever episode of BCT through this WMHP and had clinical elevations on the PHQ-9 or GAD-7 at the start of care. Participants were included regardless of episode completion status, including those who dropped out of care. Clinical exclusion criteria are described in previous publications involving this BCT program and in Supplementary Methods Section (Lungu et al., 2020). To be included in the current study, individuals needed to have completed a valid baseline assessment (e.g., PHQ-9 and GAD-7) of symptoms, 1 additional assessment while in care initially, and were eligible to be sent a 3, 6, 9, or 12-month follow-up assessment. See Supplementary Information for additional assessment requirements for inclusion and patient selection methods.

**Fig. 1:**
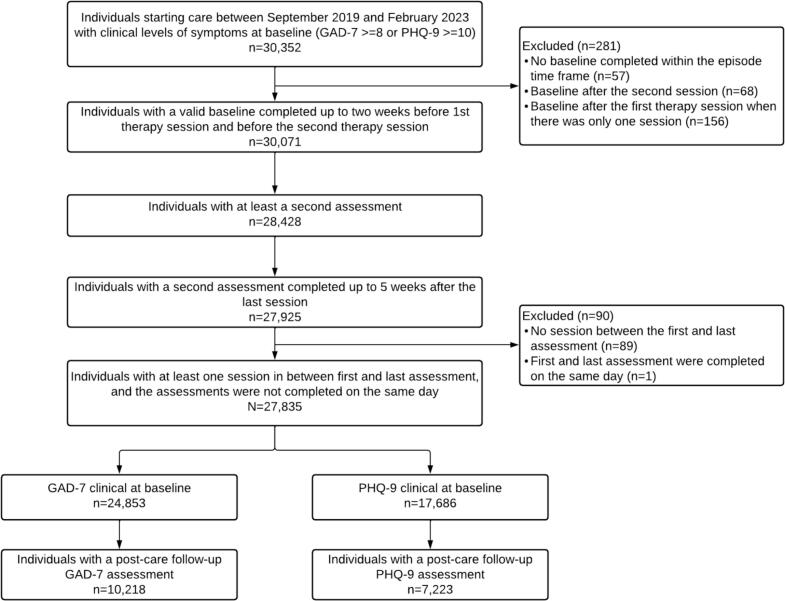
Participant flow diagram.

The CONSORT diagram illustrating the stepwise exclusion of clients is illustrated in Fig. 1. Exclusion criteria were mirrored from prior analyses on samples from this population (Lungu et al., 2020; Owusu et al., 2022).

## 4 Statistical analysis

Group differences between subgroups of clients used in analysis were examined on demographic characteristics, baseline and final in-episode symptom assessments, and treatment duration characteristics using ANOVA test, χ^2^ independence tests, and Kruskal-Wallis Test. Reliable improvement and/or recovery from symptoms during the initial episode were computed using clients' baseline in-episode assessment as the initial score, and observed scores for the final in-episode assessment. For post-care outcomes, 3-, 6-, 9-, and 12-month FUAs and/or 2nd episode in-care assessments were used to calculate reliable improvement and/or recovery rates.

Growth curve analysis using mixed effects modeling was used to examine initial treatment episode assessment trajectories (see Supplementary Methods section), as well as trajectories of post-episode PHQ-9 and GAD-7 symptom scores collected from the FUAs. For the primary FUA analysis, clients' day of episode closure as the initial (Week = 0) score on the dependent variable, along with their available FUA scores indexed by the number of weeks between the episode closure and the completion of each FUA. This approach accounts for inter-individual differences in the response latency following an FUA prompt. Trajectories were specified using a linear base function with random client-level effects for the intercept and linear components. Additional random effects were tested at the client and provider levels but were omitted from the final models due to very small variance components (i.e., PHQ-9 ICCprovider <0.016; GAD-7 ICCprovider <0.015). Point estimates and confidence intervals for the fixed effects for the model including a provider level random intercept were practically identical to the model reported here. Clients who provided at least 1 FUA were included in the growth curve analysis, and missing data were handled using the full-information maximum likelihood estimator provided by version 1.1–35.1 of the lme4 library in R 4.3.1 (Bates et al., 2015; R Core Team, 2023).

## 5 Power

The number of clients included in the primary analyses exceeded 1000 for both the PHQ-9 and GAD-7 outcomes. A sufficiently large number of clients (N_PHQ-9_ = 4453; N_GAD-7_ = 6287) provided 2 or more FUAs for the post-care model, and the overall number of responses were obtained for each FUA to reliably estimate the growth function. Observed sample size for each FUA is provided in Table 3. Prior simulation work indicates that the full-information estimation approach used in the primary analysis provides minimally biased estimates of the growth function with the observed levels of missingness under the conditional missing-at-random assumption (Hedeker and Gibbons, 2006). For all analyses, alpha was set at 0.05 for determining statistical significance.

## 6 Results

### 6.1 Participant characteristics and rates of reliable improvement and/or recovery

Demographic and symptom characteristics of eligible clients who engaged in an initial treatment episode of BCT are described in Supplementary Table 1, along with observed rates of reliable improvement and recovery for the initial episode of care in Supplementary Table 2. The values observed in the present sample were consistent with prior published evaluations of BCT (Owusu et al., 2022), with rates of reliable improvement or recovery for people with clinical anxiety or depression symptoms around 88 %, suggesting high levels of initial treatment success. In the year following treatment, approximately 21 % of clients returned for additional individual therapy (21.0 % for depression, 21.6 % for anxiety; AT) and 4 % received additional booster sessions (4.4 % for depression and anxiety; AB).

Demographic characteristics of all clients presenting with clinically significant depression who also responded to at least one FUA (*N* = 7223) are provided in Table 1, along with group specific values for clients who received No Additional Therapy (NAT), those who engaged with Additional Therapy (AT), and those receiving an Additional Booster (AB). Clients who return to care (AT or AB groups) were provided with more opportunities to provide FUA as part of their subsequent in-care assessments, thereby increasing the likelihood that these clients were included in the FUA sample. χ2 independence tests and ANOVA indicated significant group differences with those who received no additional therapy being older in age, a higher proportion male, and a different distribution in race/ethnicity. Group differences were also observed for the final in-episode PHQ-9 and GAD-7 scores, with those receiving additional therapy having higher PHQ-9 or GAD-7 scores at the end of care and greater number of sessions attended. Table 2 illustrates a similar pattern of differences among clients with clinically significant anxiety (*N* = 10,218) who responded to at least 1 FUA across the NAT, AT, and AB groups, with those receiving no additional therapy being older, higher proportion male, and having a different distribution in race/ethnicity. Finally, clients in the AT group reported higher final in-episode PHQ-9 and GAD-7 scores, attended more therapy sessions, and had a longer episode duration (GAD-7 only). The effect size estimates associated with the observed cross-group differences were small in magnitude. Differences in groups by FUA completion status are also reported in Supplementary Table 3.

**Table 1:** Participant Characteristics of the 3 post-care engagement groups with FUAs and clinical PHQ-9.

		Entire Sample	No Additional Therapy	Additional Therapy	Additional Booster	Difference Test (*p*-value)	Effect size
		N = 7223	*n* = 3176 (44.0 %)	*n* = 3392 (47.0 %)	*n* = 655 (9.1 %)		
Age, mean (SD)		33.76 (9.50)	35.11 (10.08)	32.69 (8.88)	32.78 (8.92)	58.21 (<0.001)	0.016
Gender, n (%)	*Female*	4891 (67.71)	2077 (65.40)	2364 (69.69)	450 (68.70)	25.48 (<0.001)	0.042
	*Male*	2202 (30.49)	1056 (33.25)	957 (28.21)	189 (28.85)		
	*Something else or missing*	130 (1.80)	43 (1.35)	71 (2.09)	16 (2.44)		
Race/Ethnicity, n (%)	*Asian or Pacific Islander*	1412 (19.55)	570 (17.95)	723 (21.31)	119 (18.17)	24.04 (0.02)	0.041
	*Black or African American*	628 (8.69)	279 (8.78)	282 (8.31)	67 (10.23)		
	*Hispanic or Latino*	748 (10.36)	331 (10.42)	351 (10.35)	66 (10.08)		
	*Multiple*	658 (9.11)	295 (9.29)	309 (9.11)	54 (8.24)		
	*Other*	152 (2.10)	75 (2.36)	64 (1.89)	13 (1.98)		
	*White*	3477 (48.14)	1575 (49.59)	1580 (46.58)	322 (49.16)		
	*Prefer not to disclose or missing*	148 (2.05)	51 (1.61)	83 (2.45)	14 (2.14)		
Baseline PHQ-9, mean (SD)		14.50 (3.71)	14.46 (3.71)	14.52 (3.71)	14.51 (3.75)	0.22 (0.806)	
Baseline GAD-7, mean (SD)		12.42 (4.70)	12.32 (4.72)	12.48 (4.69)	12.59 (4.66)	1.36 (0.256)	
Final In-Care PHQ-9, mean (SD)		6.06 (4.97)	5.38 (4.86)	6.68 (5.04)	6.18 (4.71)	57.09 (<0.001)	0.016
Final In-Care GAD-7, mean (SD)		5.75 (4.50)	5.08 (4.36)	6.38 (4.55)	5.78 (4.38)	70.02 (<0.001)	0.019
# Therapy sessions completed, median [Q1,Q3]		7.00 [5.00,10.00]	7.00 [5.00,9.00]	8.00 [5.00,10.00]	7.00 [5.00,9.00]	19.91 (<0.001)	0.003
Duration of care (weeks), median [Q1,Q3]		9.71 [5.57,14.14]	9.71 [6.00,14.00]	9.86 [5.29,14.86]	9.29 [5.00,13.86]	5.55 (0.062)	

Notes. PHQ-9: 9-item Patient Health Questionnaire; GAD-7: 7-item Generalized Anxiety Disorder scale; FUA: Follow-up assessment. Tests: ANOVA for Age, Baseline PHQ-9/GAD-7, Final In-Care PHQ-9/GAD-7; Chi Square for Gender and Race/Ethnicity. Kruskal-Wallis Test for # Therapy sessions completed and Duration of care (weeks). Effect sizes: Eta squared for Age, Baseline PHQ-9/GAD-7, Final In-Care PHQ-9/GAD-7; Cramer's V for Gender and Race/Ethnicity. Epsilon squared for # Therapy sessions completed and Duration of care (week).

**Table 2:** Participant Characteristics of the 3 post-care engagement groups with FUAs and clinical GAD-7.

		Entire Sample	No Additional Therapy	Additional Therapy	Additional Booster	Difference Test (p-value)	Effect size
		N = 10,218	*n* = 4422 (43.3 %)	*n* = 4860 (47.6 %)	*n* = 936 (9.2 %)		
Age, mean (SD)		33.60 (9.29)	35.07 (9.88)	32.48 (8.63)	32.42 (8.73)	99.97 (<0.001)	0.019
Gender, n (%)	*Female*	7000 (68.51)	2910 (65.81)	3435 (70.68)	655 (69.98)	38.40 (<0.001)	0.043
	*Male*	3065 (30.00)	1458 (32.97)	1348 (27.74)	259 (27.67)		
	*Something else or missing*	153 (1.50)	54 (1.22)	77 (1.58)	22 (2.35)		
Race/Ethnicity, n (%)	*Asian or Pacific Islander*	2004 (19.61)	789 (17.84)	1032 (21.23)	183 (19.55)	34.04 (0.001)	0.041
	*Black or African American*	806 (7.89)	370 (8.37)	370 (7.61)	66 (7.05)		
	*Hispanic or Latino*	1028 (10.06)	474 (10.72)	461 (9.49)	93 (9.94)		
	*Multiple*	913 (8.94)	376 (8.50)	451 (9.28)	86 (9.19)		
	*Other*	229 (2.24)	97 (2.19)	113 (2.33)	19 (2.03)		
	*White*	5035 (49.28)	2247 (50.81)	2318 (47.70)	470 (50.21)		
	*Prefer not to disclose or missing*	203 (1.99)	69 (1.56)	115 (2.37)	19 (2.03)		
Baseline PHQ-9, mean (SD)		11.20 (5.39)	11.24 (5.38)	11.17 (5.39)	11.18 (5.43)	0.19 (0.827)	
Baseline GAD-7, mean (SD)		12.90 (3.62)	12.84 (3.60)	12.94 (3.64)	12.89 (3.66)	0.85 (0.426)	
Final In-care PHQ-9, mean (SD)		5.13 (4.71)	4.53 (4.51)	5.68 (4.88)	5.08 (4.38)	69.25 (<0.001)	0.013
Final In-care GAD-7, mean (SD)		5.59 (4.34)	4.94 (4.17)	6.19 (4.45)	5.59 (4.18)	96.37 (<0.001)	0.019
# Therapy sessions completed, median [Q1,Q3]		7.00 [5.00,10.00]	7.00 [5.00,9.00]	8.00 [5.00,10.00]	7.00 [5.00,9.00]	36.85 (<0.001)	0.004
Duration of care (weeks), median [Q1,Q3]		9.86 [5.86,14.25]	9.57 [6.00,13.86]	10.00 [5.57,15.00]	9.43 [5.29,14.29]	7.52 (0.023)	0.001

Notes. PHQ-9: 9-item Patient Health Questionnaire; GAD-7: 7-item Generalized Anxiety Disorder scale; FUA: Follow-up assessment. Tests: ANOVA for Age, Baseline PHQ-9/GAD-7, Final In-Care PHQ-9/GAD-7; Chi Square for Gender and Race/Ethnicity. Kruskal-Wallis Test for # Therapy sessions completed and Duration of care (weeks). Effect sizes: Eta squared for Age, Baseline PHQ-9/GAD-7, Final In-Care PHQ-9/GAD-7; Cramer's V for Gender and Race/Ethnicity. Epsilon squared for # Therapy sessions completed and Duration of care (week).

For clients in the NAT group, reliable improvement and/or recovery rates at 3-, 6-, 9-, and 12-month FUA are provided in Table 3 (others in Supplementary Table 4). Rates are provided separately for clinical depression, clinical anxiety, or clients with either clinical elevation at baseline. Reliable improvement, recovery, improvement and recovery, as well as improvement or recovery rates for clients with clinical depression, and/or clinical anxiety were consistently highest at the 3-month FUA (67.6 % to 85.7 %) and showed small declines as the follow-up interval increased. Rates of reliable improvement or recovery for those with either clinical anxiety or depression (the most inclusive picture of clinical improvement) were generally higher and ranged from 81.2 % to 85.7 % across all follow-up periods.

**Table 3:** Rates of reliable improvement, recovery, or relapse on the PHQ-9 and GAD-7 for those who did not receive additional WMHP therapy.

Follow-up Time Period	Sample Size	Reliable Improvement^a^	Recovery^b^	Reliable Improvement and Recovery^c^	Reliable Improvement or Recovery^d^
**Clinical Depression**					
3-month FUA	1610	72.86 %	77.27 %	68.07 %	82.05 %
6-month FUA	1035	67.54 %	74.78 %	64.15 %	78.16 %
9-month FUA	742	64.42 %	72.51 %	60.38 %	76.55 %
12-month FUA	310	65.16 %	72.58 %	63.23 %	74.52 %
**Clinical Anxiety**					
3-month FUA	2282	76.51 %	73.62 %	67.57 %	82.56 %
6-month FUA	1466	72.44 %	71.83 %	64.87 %	79.40 %
9-month FUA	1028	71.40 %	69.07 %	62.94 %	77.53 %
12-month FUA	439	72.21 %	70.62 %	63.10 %	79.73 %
**Clinical Depression or Anxiety**					
3-month FUA	2555	78.71 %	80.95 %	71.90 %	85.71 %
6-month FUA	1647	75.17 %	77.79 %	69.58 %	82.94 %
9-month FUA	1170	73.42 %	76.08 %	66.92 %	81.20 %
12-month FUA	494	75.30 %	76.63 %	69.03 %	81.98 %

Notes. PHQ-9: 9-item Patient Health Questionnaire; GAD-7: 7-item Generalized Anxiety Disorder scale.

a Reliable Improvement: ≥ 4 decrease on the FUA GAD-7 among those with baseline GAD-7 ≥ 8 and/or ≥ 6 decrease on the FUA PHQ-9 among those with baseline PHQ-9 ≥ 10

b Recovery: FUA GAD-7 < 8 among those with baseline GAD-7 ≥ 8 and/or FUA PHQ-9 < 10 among those with baseline PHQ-9 ≥ 10

c Reliable Improvement and Recovery: ≥ 4 decrease on the FUA GAD-7 and FUA GAD-7 < 8 among those with baseline GAD-7 ≥ 8; and/or, ≥ 6 decrease on the FUA PHQ-9 and FUA PHQ-9 < 10 among those with baseline PHQ-9 ≥ 10

d Reliable Improvement or Recovery: ≥ 4 decrease on the FUA GAD-7 or FUA GAD-7 < 8 among those with baseline GAD-7 ≥ 8; and/or, ≥ 6 decrease on the FUA PHQ-9 or FUA PHQ-9 < 10 among those with baseline PHQ-9 ≥ 10.

### 6.2 Growth-curve analysis of initial treatment episode and post-treatment follow-up assessments

Growth curve analysis of the outcomes in the initial treatment episode are presented in Supplementary Table 5. Results are similar to previous publications (Owusu et al., 2022) for Model 1 for PHQ-9 and GAD-7 scores, indicating a downward trajectory of symptoms for each week in treatment that tapers over time. Model 2 with interaction terms highlights the differences in initial treatment trajectories for groups who had different post-care engagement behaviors (AT, NAT, AB), providing additional support for examining post-care outcomes across the separate groups.

The leftmost side of Table 4 contains the fixed effects of the growth curve analysis for the clients with clinically elevated depression scores at baseline using all available PHQ-9 FUAs. Results from Model 1 suggest that on average clients reported subclinical levels of depression at the time of treatment episode closure (b_0_ = 6.60), and the average trajectory across all groups suggested an approximately b_1_ = 0.12 units weekly increase in PHQ-9 post-treatment, which became flatter over time (b_2_ = −0.002). Model 2 incorporated dummy variables identifying clients in the Additional Therapy (AT) and Additional Booster (AB) groups (NAT as reference category), along with the corresponding interaction terms involving Week and Week^2^. The first-order coefficients for the Intercept (b_0_ = 5.56), Week, and Week^2^ terms reveal that the NAT group exhibited an initial increase of b_1_ = 0.03 units in PHQ-9 post-treatment, which remained approximately linear over the course of the follow-up period, suggesting an approximately 1.6 point increase over 12-months for those receiving no additional therapy (NAT). The first-order effect of the AT dummy variable suggests that these clients reported higher depression at the close of their initial episode (b_3_ = 1.96), and the significant Week×AT interaction (b_5_ = 0.11) suggests that clients who received additional therapy (AT) exhibited a steeper initial increase in symptoms, but the significant Week^2^ × AT interaction (b_7_ = −0.003) indicates that the initial increase in symptoms decelerated over time. The coefficients involving the AB dummy variable indicate that clients receiving additional booster session(s) (AB) reported higher PHQ-9 scores at the close of their initial treatment episode (b_4_ = 1.17). The significant Week×AB interaction suggests that these clients exhibited stronger initial increases in symptoms (b_6_ = 0.11), and the Week^2^ × AB interaction (b_8_ = −0.002) indicates that the increase in depression decelerated over time.

**Table 4:** Growth Curve Model Results of Follow-up Assessments of Depression and Anxiety Symptoms.

	Clinical Depression		Clinical Anxiety	
	Model 1: Without post-care engagement groups	Model 2: Interaction with post-care engagement groups	Model 1: Without post-care engagement groups	Model 2: Interaction with post-care engagement groups
Intercept	6.60 (6.48, 6.72)	5.56 (5.38, 5.73)	6.10 (6.01, 6.19)	5.10 (4.97, 5.23)
	*t* = 110.38***	*t* = 62.63***	*t* = 140.03***	*t* = 78.42***
Week	0.12 (0.11, 0.13)	0.03 (0.02, 0.05)	0.12 (0.11, 0.12)	0.04 (0.03, 0.05)
	*t* = 26.53***	*t* = 3.89***	*t* = 33.02***	*t* = 6.65***
Week^2^	−0.002 (−0.002, −0.002)	0.0003 (−0.0001, 0.001)	−0.002 (−0.002, −0.002)	−0.0000 (−0.0003, 0.0003)
	*t* = −20.34***	*t* = 1.51	*t* = −25.13***	*t* = −0.32
Additional Therapy		1.96 (1.72, 2.20)		1.86 (1.68, 2.03)
		*t* = 16.09***		*t* = 20.93***
Additional Booster		1.17 (0.75, 1.58)		1.10 (0.79, 1.40)
		*t* = 5.48***		*t* = 7.10***
Week*Additional Therapy		0.11 (0.09, 0.13)		0.09 (0.07, 0.10)
		*t* = 11.05***		*t* = 11.38***
Week*Additional Booster		0.11 (0.07, 0.15)		0.12 (0.09, 0.15)
		*t* = 5.75***		*t* = 8.03***
Week^2^*Additional Therapy		−0.003 (−0.004, −0.003)		−0.003 (−0.003, −0.002)
		*t* = −13.82***		*t* = −14.25***
Week^2^*Additional Booster		−0.002 (−0.003, −0.001)		−0.002 (−0.003, −0.001)
		*t* = −3.57***		*t* = −5.37***
Log Likelihood	−100,368.40	−100,061.90	−135,215.60	−134,759.20
Akaike Inf. Crit.	200,750.90	200,149.80	270,445.30	269,544.50
Bayesian Inf. Crit.	200,809.90	200,259.40	270,506.60	269,658.40

Note:*p *<* .05, ** p < .01, ***p *<* *.001*

Clinical Depression: random client-level effects for the intercept and linear components; Clinical Anxiety: random client-level effects for the intercept and linear components.

The right hand columns of Table 4 contain the results for clients with clinically elevated anxiety symptoms at baseline. Results from Model 1 indicate that on average, clients reported subclinical levels of anxiety at the time of treatment episode closure (b_0_ = 6.10), and the average trajectory suggested an approximately b_1_ = 0.12 units weekly increase in GAD-7 post-treatment that became flatter over time (b_2_ = −0.002). Turning to Model 2, the group receiving no additional therapy (NAT) reported subclinical levels of anxiety at the time of treatment episode closure (b_0_ = 5.10) and exhibited an average increase in GAD-7 scores of approximately b_1_ = 0.04 units per week that remained linear throughout the follow-up interval, suggesting an approximately 2.1 point increase over 12-months. The first-order effect of the AT dummy variable (b_3_ = 1.86) suggests that the group who received additional therapy (AT) reported significantly higher levels of anxiety at the close of their initial episode, whereas the Week×AT interaction (b_5_ = 0.09) signals a steeper initial change for this group, but the Week^2^ × AT interaction (b_7_ = −0.003) indicates that the trajectory for this group becomes flatter over time. Finally, the first-order effect of the AB dummy variable indicates that clients who received additional booster session(s) (AB) reported higher anxiety at the close of their initial episode (b_4_ = 1.10) and the significant Week×AB interaction (b_6_ = 0.12) suggests that clients in this group showed a larger initial increase in anxiety symptoms (relative to the NAT group), whereas the significant Week^2^ × AB interaction (b_8_ = −0.002) indicates that the initial increase in symptoms flattens over time. Plots of the predicted trajectories for results in Table 4 are provided in the Supplementary Figs. 1 and 2. Information criteria statistics indicated that Model 2 provided a better fit to the data (relative to Model 1) for both the PHQ-9 and GAD-7 analysis. Sensitivity analysis incorporating demographic and baseline clinical characteristics resulted in a similar pattern of findings. Additionally, sensitivity analyses evaluating the impact of characteristics unique to those who did and did not complete FUA on clinical outcomes trajectories are described in the Supplementary Table 6 and were similar to the findings reported in the primary analysis.

## 7 Discussion

### 7.1 Average persistence of rates of reliable change and recovery

The present study reported depression and anxiety symptoms from clients at 3-, 6-, 9-, and 12-months following engagement with a blended care therapy program. Rates of reliable improvement or recovery during the initial treatment episode were nearly identical to previous evaluations of this program (Owusu et al., 2022), supporting successful scaling of the WMHP. More than 82 % of clients who initially had clinical elevations of depressive symptoms exhibited reliable improvement or recovery in symptoms between the baseline and final assessment during their treatment episode. For individuals who received no additional therapy in the year following their initial treatment episode, reliable improvement or recovery rates from baseline levels of depression severity were between 75 and 82 % among observed respondents at each post-episode follow-up occasion.

A similar pattern of reliable improvement or recovery was observed among clients with clinical elevations of anxiety symptoms, with >84 % showing reliable improvement or recovery during their primary treatment episode, and observed post-episode reliable improvement or recovery rates were between 78 and 83 % among observed respondents over the different follow-up periods. While differences in measurement methods and definitions of improvement or recovery make direct comparisons difficult, rates of reliable improvement or recovery were much higher than those seen in meta-analyses for depression (Cuijpers et al., 2021) and anxiety (Loerinc et al., 2015) where individuals were treated with evidence-based treatments in highly controlled clinical trials.

### 7.2 Modeling results

Results from the growth curve analyses revealed that clients who received no additional therapy exhibited modest linear increases of approximately 1.6 points on the PHQ-9 and 2.1 points on the GAD-7 between the end of their active treatment episode and the end of the 12-month follow-up period. However, these increases in depression and anxiety symptoms are not clinically meaningful and smaller than the reliable change indices for each of these measures of 6 and 4 points, respectively, indicating these changes are not significant (Liness et al., 2019). These small symptom changes are also dwarfed by the magnitude of symptom reduction observed during the typical 8 to 9-week active treatment episode, with a decrease of approximately 8 scale units for the PHQ-9 and 7 scale units for the GAD-7. A slight upward trend in symptoms for those with previous diagnoses of anxiety or depression in the year following treatment has been demonstrated in longitudinal clinical trials research (Sæther et al., 2019) and could represent a natural course of anxiety and depression symptoms over time. An additional longitudinal examination of diagnostic trajectories for individuals with anxiety and depression indicates that most individuals experience intermittent recovery and recurrence of diagnoses in the years after diagnosis (Penninx et al., 2011; Solis et al., 2021), further highlighting the importance of ongoing monitoring and additional research in this area. Future research would also benefit from the inclusion of measures of functioning to evaluate how symptom fluctuations impact everyday functioning, particularly post-care.

All clients provided at least a second assessment during their initial treatment episode, which provided the first PHQ-9 or GAD-7 score used in the post-care growth curve analysis. In addition, all clients in the growth curve analysis analyzing post-care outcomes responded to at least 1 FUA, and many responded to 2 or more. Nevertheless, there was a significant amount of missing data and small differences between groups who did and did not complete FUAs. These small differences included slightly higher final in-care PHQ-9 (0.63 points) and GAD-7 (0.49) scores for the respective clinical group, fewer completed sessions (1 fewer), and shorter duration of care (around 2 fewer weeks) for those who did not complete FUAs. Sensitivity analyses support that FUA missingness and related participant characteristics did not meaningfully impact the primary study conclusions; however, future research is encouraged to further evaluate the differences between those who respond to FUAs in naturalistic studies, given the lack of comparative data in this area.

### 7.3 Return to care

An additional important finding of this study was the percentage of individuals who returned for additional individual therapy the year following treatment completion, which occurred at a rate of approximately 21 %. As is the practice in most evidence-based forms of treatment (Barlow et al., 2017; Hayes et al., 2011; Hofmann et al., 2010; Linehan, 2014), final sessions of this BCT program focused on supporting clients in maintaining skill usage, monitoring progress, and increasing awareness of when clients should potentially return to care. Individuals who returned to care were easily tracked via the care platform, providing granular data on client behavior, care-seeking, and outcomes for this large cohort over time. In the only identified study using data from the United States in the Veterans Affairs Healthcare system, 77 % of veterans who completed an evidence-based treatment program returned for additional individual psychotherapy in the year following care, suggesting return to care rates may be much higher in other systems (Baier et al., 2024). When individuals completed FUAs, consistent with routine outcome monitoring recommendations, they received feedback on their total scores and were provided with information on how to re-engage in care, if indicated and desired. It is unknown if this feedback may have impacted behavior, such that individuals who had clinical elevations and were informed of their scores then returned to care at greater rates. Although there were some demographic and treatment differences between those who returned to care and those who did not, these effect sizes were all small. Future research should examine predictors of return to care to help identify those who would benefit most from ongoing follow-up or more intentional long-term support after completion of the first treatment episode.

Little is known about long-term stability of treatment gains at a symptom level, with relapse rates being the closest approximation of this outcome in the literature. Long-term relapse rates are also rarely reported, even in RCTs. Reported relapse rates in depression following treatment with EBT are typically from very small sample sizes using variable definitions of relapse (e.g., return of symptoms to clinical levels, re-diagnosed), with rates varying from 34 % to 50 % in the years following treatment (Chen et al., 2022; Wojnarowski et al., 2019). Rates of relapse are even less frequently reported for anxiety, with 1 to 4 year relapse rates ranging from 13 to 42 % (Lorimer et al., 2021), and many of these studies also faced similar challenges of definition and very small sample sizes. Data from longitudinal studies suggest that both depression and anxiety disorders recur at high rates, with 79 % of individuals experiencing recurrence over the course of 9 years (Solis et al., 2021). These data and other care models focused on chronic care (Bauer et al., 2019) suggest that a return to care for a certain percentage of clients is not only expected, but should be anticipated and planned for in the care model with ongoing monitoring and an easy pathway to re-engaging with treatment when issues arise.

Although the reasons for returning to care are unknown in this study (e.g., relapse, new treatment need or diagnosis), the rate at which individuals returned for additional therapy were notably lower than many of the return to care rates seen in other studies (Baier et al., 2024; Sæther et al., 2019). It is important to note however that a variety of factors aside from recurrence could lead individuals to return to care, including new life stressors or presentation of new symptoms not previously addressed in care. Individuals returning to seek care through the same WMHP is a positive signal of treatment feasibility and acceptability, as well as potential effectiveness. It is also encouraging that those affected by ongoing or increasing depressive and anxiety symptoms may have returned to seek care for what are likely chronic mental health concerns, as timely re-engagement in treatment may prevent functional impairments from increasing (Ten Have et al., 2022). Future research should investigate the impact of returning to care on the duration of treatment and timing of subsequent recovery.

### 7.4 Strengths and limitations

This pragmatic study evaluates clinical outcomes in the largest known published sample of individuals treated with BCT with long-term follow-up data. It also reports on outcomes in naturalistic care with real-world data, increasing generalizability of results to everyday mental health practice. Clients in the study came from a wide range of racial and ethnic backgrounds, with only around 50 % of individuals identifying as White, which is more representative of the United States population than samples typically included in clinical trials (Polo et al., 2019). This naturalistic study design does however limit the ability to make strong statements in comparison to a different form of treatment (e.g., traditional synchronous therapy only), such as would be possible with an RCT. Given the naturalistic design of this study, participants were not financially incentivized to complete follow-up assessment measures, resulting in more missing data and lower overall response rates than are typically seen in RCTs. Though effect sizes were very small, there were differences in characteristics of those who responded to follow-up assessments (FUAs) and those who did not, which may limit the generalizability of these results. Future naturalistic research studies should explore methods to increase responding to FUA in routine care. The sample also included individuals who were provided services through an employer-sponsored program, representing an employed population or their dependents whose stressors and access to mental health care may differ from others, including those who do not work, are unemployed, or retired. Future research should explore the effectiveness of a BCT program in a sample of individuals with varying employment statuses to enhance applicability of these findings.

## 8 Conclusions

This study demonstrated strong clinical outcomes for those who engaged in an episode of BCT, with high rates of reliable improvement or recovery in their initial episode of care, replicating previous findings from this program with a substantially larger sample. This study is also the first at this scale to show strong and stable long-term clinical improvements in anxiety and depression symptoms in the year following treatment for individuals initially treated with a BCT program who responded to follow-up assessments and did not return for additional therapy. Return to care rates in the year following treatment were also relatively low. Future research should continue to describe the longitudinal trajectories of anxiety and depressive symptoms following treatment with a BCT program. For instance, collecting larger and more clinically diverse samples would allow future studies to identify factors associated with persistence in symptom remission or relapse, such as levels of engagement with digital therapy tools during and following treatment. Identifying these prognostic indicators can guide the development of more adaptive, and in turn, effective treatment protocols.

## Abbreviations

BCT: blended care therapy
CBT: cognitive-behavioral therapy
EBT: evidence-based therapy
RCT: randomized controlled trial
PHQ-9: Patient Health Questionnaire-9
GAD-7: Generalized Anxiety Disorder-7

## CRediT authorship contribution statement

Conceptualization (Ideas; formulation or evolution of overarching research goals and aims.) JLL, AL, CEC, MSW

Data curation (Management activities to annotate (produce metadata), scrub data and maintain research data (including software code, where it is necessary for interpreting the data itself) for initial use and later re-use.) SYC, PW

Formal analysis (Application of statistical, mathematical, computational, or other formal techniques to analyze or synthesize study data.) SYC, REW, PW

Funding acquisition (Acquisition of the financial support for the project leading to this publication.) CEC

Investigation (Conducting a research and investigation process, specially performing the experiments, or data/evidence collection.) JLL, AL, SYC, REW, PW

Methodology (Development or design of methodology; creation of models.) JLL, SYC, REW, PW, AL

Project administration (Management and coordination responsibility for the research activity planning and execution.) JLL

Resources (Provision of study materials, reagents, materials, patients, laboratory samples, animals, instrumentation, computing resources, or other analysis tools.) CEC, AAV

Software (Programming, software development; designing computer programs; implementation of the computer code and supporting algorithms; testing of existing code components.) SYC, REW, PW

Supervision (Oversight and leadership responsibility for the research activity planning and execution, including mentorship external to the core team.) JLL, AL

Validation (Verification, whether as a part of the activity or separate, of the overall replication/reproducibility of results/experiments and other research outputs.) n/a

Visualization (Preparation, creation and/or presentation of the published work, specifically visualization/data presentation.) SYC

Writing – original draft (Preparation, creation and/or presentation of the published work, specifically writing the initial draft (including substantive translation).) JLL, SYC, REW, PW, AL, CEC, AAV

Writing – review & editing (Preparation, creation and/or presentation of the published work by those from the original research group, specifically critical review, commentary or revision – including pre- or post-publication stages.) JLL, SYC, REW, PW, AL, CEC, AAV, MSW

## Funding

The study was funded by Lyra Health. The funder of the study was involved in study design, data interpretation, and writing of the report, and informed during data collection and data analysis. The corresponding author had full access to all of the data in the study and had final responsibility for the decision to submit for publication.

## Code availability

All relevant software and code used for analysis are cited within the manuscript or supplementary materials.

## Declaration of competing interest

JLL, SYC, and PW are currently or were previously employed by Lyra Health, receive income from Lyra Health, and have been granted equity in Lyra Health. CEC, AAV, MSW, and AL are employed by Lyra Health and Lyra Clinical Associates, receive income from Lyra Health and Lyra Clinical Associates, and have been granted equity in Lyra Health. REW is a paid consultant for Lyra Health.

## Data Availability

The datasets generated during and/or analyzed during the current study are not publicly available due to the fact that the data is related to the delivery of health care and subject to the Health Information Portability and Accountability Act (HIPAA) of 1996. As a business associate under HIPAA, Lyra is only permitted to use patient information as outlined in our Business Associate Agreements (BAAs) with our customers, which are covered entities. Many of our customer agreements do not permit us to share de-identified patient data; given this, we are unable to share the de-identified data due to contractual restrictions.

## Appendix A Supplementary data

supplementary material Image 1
