# Supplementary material for: Clinical outcomes from blended care therapy for anxiety and depression in the year after treatment
Source: Internet Interv. 2024 Dec 28;39:100798. doi: 10.1016/j.invent.2024.100798 (PMC11743894; doi:10.1016/j.invent.2024.100798)
Supplement: Supplementary file 1 — supplementary material [file mmc1.docx]

## Supplementary Materials

**Supplementary Methods Section**

*Patient Population*

Participants with the following characteristics were excluded from the blended care therapy program described in this paper (Lungu et al., 2020): preference for in-person therapy, under 18 years of age, active suicidality, active homicidality, a current diagnosis of a mental health disorder with psychotic features not stabilized on medications, unstable bipolar disorder, or a current diagnosis of severe alcohol or substance use disorder requiring inpatient treatment.

*Assessment Inclusion Criteria and Analysis*

To be considered valid, baseline assessments needed to be completed within 2 weeks of the first therapy session, and before a second session occurred. Individuals were also excluded if baseline assessments were completed after the first session and a second session did not occur. A second in-care assessment needed to be completed on a different day than the baseline and at a maximum of 5 weeks after the last therapy session. A proportion of participants and assessments described in the initial treatment episode analyses are also represented in a prior publication (Owusu et al., 2022).

Participants were considered eligible for follow-up assessments (FUAs) if at least 90 days had elapsed since their episode close date. FUAs were categorized according to the number of days that had passed since the initial treatment episode had been closed. The following categories were used: a) short term follow-up: days 36-89 days; b) 3-month follow-up: 90-179 days, c) 6-month follow-up: 180-269 days, d) 9-month follow-up: 270-359 days; e) 12-month follow-up: 360-374 days. When more than 1 assessment was completed within a given follow-up interval, assessments were averaged to generate a mean score for use in calculating reliable improvement and/or recovery rates. Individuals who engaged in additional therapy or a booster were sent additional in-care assessments during their second treatment episode, which means they represent a larger proportion in those with FUAs.

For a small percentage of participants, there was a substantial period of time between the date of their last session and the official closure of their episode in the records (considered Day 0 for follow-up). Approximately 90% of episodes were closed within 5 weeks of the last session, thus this was chosen as an imposed end date for those with substantial time gaps between last session and episode closures in the records to begin calculating Day 0 for the follow-up time period.

*Statistical Analysis Plan for Initial In-Care Growth Curve Analysis*

Growth curve-analysis and mixed effects models were used to examine the growth trajectories of PHQ-9 and GAD-7 symptom scores during the initial treatment episode using clients’ first therapy session as the initial (Week=0) score on the dependent variable, along with their available in-care assessments indexed by the number of weeks between the first session and the completion of each in-care assessment. Model 1 included fixed-effects terms for linear (Week) and quadratic (Week^2^) time, which describe the average trajectory of symptoms. Clients’ post-care engagement behaviors (No additional therapy: NAT, Additional individual therapy: AT, Additional booster session: AB) were added as predictors to Model 2, along with the corresponding interaction terms with Week and Week^2^ to evaluate potential cross-group differences. This approach allows for obtaining average trajectories of the assessments while accounting for individual-level variability. All models estimated random effects for the intercept, linear, and quadratic terms at the client level. Additionally, random effect for the intercept term at the provider level was estimated for participants with clinical elevations of anxiety symptoms at baseline. Clients with a valid second in-care assessment were included in the growth curve analysis, and missing data was handled using the full-information maximum likelihood estimator provided by version 1.1-35.1 of the lme4 library in R 4.3.1 (Bates et al., 2015; R Core Team, 2022).

**Supplementary Results Section**

The Supplementary Table 1 below includes descriptive statistics of demographic and clinical characteristics of those who initiated an episode of blended care therapy (BCT) and met study criteria. The PHQ-9 Clinical sample includes all those who had clinical elevations on the PHQ-9 and the GAD-7 Clinical sample includes all those who had clinical elevations on GAD-7.

| **Supplementary Table 1. Participant characteristics of those eligible for inclusion in in-care outcome models** | | | | |
| --- | --- | --- | --- | --- |
|  |  | **Entire sample** | **PHQ-9 Clinical** | **GAD-7 Clinical** |
|  |  | n = 27,835 | n = 17,686 | n = 24,853 |
| **Age, mean (SD)** | | 33.31 (9.18) | 33.30 (9.35) | 33.26 (9.14) |
| **Gender, n (%)** | *Female* | 18169 (65.27) | 11489 (64.96) | 16292 (65.55) |
|  | *Male* | 9240 (33.20) | 5883 (33.26) | 8182 (32.92) |
|  | *Something else or missing* | 426 (1.53) | 314 (1.78) | 379 (1.52) |
| **Race/Ethnicity, n (%)** | *Asian or Pacific Islander* | 5398 (19.39) | 3347 (18.92) | 4806 (19.34) |
|  | *Black or African American* | 2120 (7.62) | 1460 (8.26) | 1875 (7.54) |
|  | *Hispanic or Latino* | 2886 (10.37) | 1999 (11.30) | 2592 (10.43) |
|  | *Multiple* | 2274 (8.17) | 1511 (8.54) | 2056 (8.27) |
|  | *Other* | 559 (2.01) | 347 (1.96) | 508 (2.04) |
|  | *White* | 14046 (50.46) | 8661 (48.97) | 12520 (50.38) |
|  | *Prefer not to disclose or missing* | 552 (1.98) | 361 (2.04) | 496 (2.00) |
| **Baseline PHQ-9, mean (SD)** | | 11.47 (5.25) | 14.56 (3.77) | 11.29 (5.44) |
| **Baseline GAD-7, mean (SD)** | | 12.14 (4.22) | 12.49 (4.73) | 12.95 (3.66) |
| **Final In-Care PHQ-9, mean (SD)** | | 5.37 (4.75) | 6.43 (5.06) | 5.37 (4.80) |
| **Final In-Care GAD-7, mean (SD)** | | 5.69 (4.33) | 6.10 (4.57) | 5.88 (4.38) |
| **# Therapy sessions completed, median [Q1,Q3]** | | 7.00 [4.00,9.00] | 7.00 [4.00,9.00] | 7.00 [4.00,9.00] |
| **Duration of care (weeks), median [Q1,Q3]** | | 8.57 [4.43,13.00] | 8.43 [4.14,13.00] | 8.71 [4.43,13.00] |
| *Notes*. PHQ-9: 9-item Patient Health Questionnaire; GAD-7: 7-item Generalized Anxiety Disorder scale; FUA: Follow-up assessment | | | | |

The Supplementary Table 2 below includes rates of reliable improvement and/or recovery experienced during the initial BCT treatment episode, separated by post-care engagement groups of No Additional Therapy (NAT), Additional Therapy (AT) and Additional Booster (AB). Statistically significant differences were observed and effect sizes were overall small.

| **Supplementary Table 2: Rates of in-care reliable improvement and/or recovery on GAD-7 or PHQ-9, overall and broken down by 3 post-care engagement groups** | | | | | | |
| --- | --- | --- | --- | --- | --- | --- |
|  |  | **Post-care Engagement Group** | | |  | |
|  | **Overall** | **NAT** | **AT** | **AB** |  |  |
| **Clinical Depression** | N = 17,686 | N = 13,196 (74.6%) | N = 3,716 (21.0%) | N = 774 (4.4%) | **Difference test**  **(p-value)** | **Effect size** |
| Reliable improvement^a^ | 70.2% | 71.1% | 67% | 70.8% | 23.37 (<0.001) | 0.036 |
| Recovery^b^ | 76.4% | 76.8% | 74.7% | 78.3% | 9.03 (0.011) | 0.023 |
| Reliable improvement and recovery^c^ | 64.5% | 65.3% | 61.7% | 65.9% | 16.42 (<0.001) | 0.030 |
| Reliable improvement or recovery^d^ | 82.1% | 82.6% | 79.9% | 83.2% | 15.29 (<0.001) | 0.029 |
|  | **Overall** | **NAT** | **AT** | **AB** |  | |
| **Clinical Anxiety** | N = 24,853 | N = 18,394 (74.0%) | N = 5,366 (21.6%) | N = 1,093 (4.4%) | **Difference test**  **(p-value)** | **Effect size** |
| Reliable improvement^a^ | 78.3% | 79% | 75.5% | 79.4% | 30.71 (<0.001) | 0.035 |
| Recovery^b^ | 72.5% | 73.3% | 68.9% | 75.7% | 46.9 (<0.001) | 0.043 |
| Reliable improvement and recovery^c^ | 66.5% | 67.5% | 62.9% | 68.9% | 42.33 (<0.001) | 0.041 |
| Reliable improvement or recovery^d^ | 84.2% | 84.8% | 81.5% | 86.2% | 38.1 (<0.001) | 0.039 |
|  | **Overall** | **NAT** | **AT** | **AB** |  | |
| **Clinical Depression or Anxiety** | N = 27,835 | N = 20,614 (74.1%) | N = 5,997 (21.5%) | N = 1,224 (4.4%) | **Difference test**  **(p-value)** | **Effect size** |
| Reliable improvement^a^ | 80.9% | 81.7% | 78.1% | 81.1% | 39.69 (<0.001) | 0.038 |
| Recovery^b^ | 80.7% | 81.4% | 78.1% | 82.8% | 35.11 (<0.001) | 0.036 |
| Reliable improvement and recovery^c^ | 72.4% | 73.3% | 69% | 73.3% | 43.37 (<0.001) | 0.039 |
| Reliable improvement or recovery^d^ | 88.1% | 88.7% | 85.6% | 89.4% | 43.99 (<0.001) | 0.040 |
| *Notes*. NAT: no additional therapy; AT: additional therapy; AB: additional booster; PHQ-9: 9-item Patient Health Questionnaire; GAD-7: 7-item Generalized Anxiety Disorder scale. Chi-squared tests were used for difference tests; Cramer's V for calculating effect sizes. | | | | | | |
| a Reliable improvement: ≥ 4 decrease on the final in-care GAD-7 among those with baseline GAD-7 ≥ 8 and/or ≥ 6 decrease on the final in-care PHQ-9 among those with baseline PHQ-9 ≥ 10  b Recovery: Final-in care GAD-7 < 8 among those with baseline GAD-7 ≥ 8 and/or final in-care PHQ-9 < 10 among those with baseline PHQ-9 ≥ 10  c Reliable Improvement and Recovery: ≥ 4 decrease on the final in-care GAD-7 and final in-care GAD-7 < 8 among those with baseline GAD-7 ≥ 8; and/or, ≥ 6 decrease on the final in-care PHQ-9 and final in-care PHQ-9 < 10 among those with baseline PHQ-9 ≥ 10  d Reliable Improvement or Recovery: ≥ 4 decrease on the final in-care GAD-7 or final in-care GAD-7 < 8 among those with baseline GAD-7 ≥ 8; and/or, ≥ 6 decrease on the final in-care PHQ-9 or final in-care PHQ-9 < 10 among those with baseline PHQ-9 ≥ 10 | | | | | | |

The Supplementary Table 3 below includes descriptive statistics for demographic and clinical characteristics of those who initiated an episode of blended care therapy (BCT) and met study criteria, separated by their follow-up assessment (FUA) completion status (i.e., completed or missing). The PHQ-9 Clinical sample includes all those who had clinical elevations on the PHQ-9 and the GAD-7 Clinical sample includes all those who had clinical elevations on GAD-7. Statistically significant differences were found and all effect sizes were small.

| **Supplementary Table 3. Participant characteristics by follow-up assessments completion status, separated by PHQ-9 and GAD-7 clinical groups** | | | | | | | | | |
| --- | --- | --- | --- | --- | --- | --- | --- | --- | --- |
|  |  | **PHQ-9 Clinical** | | | | **GAD-7 Clinical** | | | |
|  |  | Completed FUA | Missing FUA | Difference test | Effect | Completed FUA | Missing FUA | Difference test | Effect |
|  |  | n = 7,223 | n = 10,463 | (p-value) | size | n = 10,218 | n = 14,635 | (p-value) | size |
| **Age, mean (SD)** | | 33.76 (9.50) | 32.98 (9.23) | 5.49 (<0.001) | 0.084 | 33.60 (9.29) | 33.03 (9.02) | 4.80 (<0.001) | 0.062 |
| **Gender, n (%)** | *Female* | 4891 (67.71) | 6598 (63.06) | 42.61 (<0.001) | 0.049 | 7000 (68.51) | 9292 (63.49) | 68.28 (<0.001) | 0.052 |
|  | *Male* | 2202 (30.49) | 3681 (35.18) |  |  | 3065 (30.00) | 5117 (34.96) |  |  |
|  | *Something else or missing* | 130 (1.80) | 184 (1.76) |  |  | 153 (1.50) | 226 (1.54) |  |  |
| **Race/Ethnicity, n (%)** | *Asian or Pacific Islander* | 1412 (19.55) | 1935 (18.49) | 22.63 (0.001) | 0.036 | 2004 (19.61) | 2802 (19.15) | 22.34 (0.001) | 0.030 |
|  | *Black or African American* | 628 (8.69) | 832 (7.95) |  |  | 806 (7.89) | 1069 (7.30) |  |  |
|  | *Hispanic or Latino* | 748 (10.36) | 1251 (11.96) |  |  | 1028 (10.06) | 1564 (10.69) |  |  |
|  | *Multiple* | 658 (9.11) | 853 (8.15) |  |  | 913 (8.94) | 1143 (7.81) |  |  |
|  | *Other* | 152 (2.10) | 195 (1.86) |  |  | 229 (2.24) | 279 (1.91) |  |  |
|  | *White* | 3477 (48.14) | 5184 (49.55) |  |  | 5035 (49.28) | 7485 (51.14) |  |  |
|  | *Prefer not to disclose/missing* | 148 (2.05) | 213 (2.04) |  |  | 203 (1.99) | 293 (2.00) |  |  |
| **Baseline PHQ-9, mean (SD)** | | 14.50 (3.71) | 14.60 (3.81) | 1.77 (0.077) |  | 11.20 (5.39) | 11.35 (5.47) | 2.20 (0.028) | 0.028 |
| **Baseline GAD-7, mean (SD)** | | 12.42 (4.70) | 12.55 (4.74) | 1.71 (0.087) |  | 12.90 (3.62) | 13.00 (3.68) | 2.14 (0.032) | 0.028 |
| **Final In-Care PHQ-9, mean (SD)** | | 6.06 (4.97) | 6.69 (5.11) | 8.16 (<0.001) | 0.124 | 5.13 (4.71) | 5.54 (4.85) | 6.80 (<0.001) | 0.087 |
| **Final In-Care GAD-7, mean (SD)** | | 5.75 (4.50) | 6.34 (4.60) | 8.48 (<0.001) | 0.129 | 5.59 (4.34) | 6.08 (4.40) | 8.67 (<0.001) | 0.112 |
| **# Therapy sessions completed, median [Q1,Q3]** | | 7.00 [5.00,10.00] | 6.00 [4.00,8.00] | 468.13 (<0.001) | 0.027 | 7.00 [5.00,10.00] | 6.00 [4.00,8.00] | 620.70 (<0.001) | 0.025 |
| **Duration of care (weeks), median [Q1,Q3]** | | 9.71 [5.57,14.14] | 7.71 [3.71,12.00] | 404.29 (<0.001) | 0.023 | 9.86 [5.86,14.25] | 7.86 [4.00,12.14] | 533.22 (<0.001) | 0.022 |
| *Notes*. PHQ-9: 9-item Patient Health Questionnaire; GAD-7: 7-item Generalized Anxiety Disorder scale; FUA: Follow-up assessment. Tests: Two sample t-test for Age, Baseline PHQ-9/GAD-7 scores, Final In-care PHQ-9/GAD-7 scores; Chi Square for Gender and Race/Ethnicity. Kruskal-Wallis Test for # Therapy sessions completed and Duration of care (weeks). Effect sizes: Hedges's g for Age, Baseline PHQ-9/GAD-7, Final In-care PHQ-9/GAD-7; Cramer's V for Gender and Race/Ethnicity. Epsilon squared for # Therapy sessions completed and Duration of care (weeks). | | | | | | | | | |

The Supplementary Table 4 below includes rates of reliable improvement and/or recovery experienced following the closure of an initial BCT treatment episode, separated by post-care engagement groups of No Additional Therapy (NAT), Additional Therapy (AT) and Additional Booster (AB). Follow-up periods are indicated by the Follow-up Time Period column.

| **Supplementary Table 4: Rates of post-care reliable improvement and/or recovery, broken down by GAD-7 and PHQ-9** | | | | | | | | | | | | | | | |
| --- | --- | --- | --- | --- | --- | --- | --- | --- | --- | --- | --- | --- | --- | --- | --- |
| **Follow-up Time Period** | **Sample Size** | | | **Reliable Improvement^a^** | | | **Recovery^b^** | | | **Reliable Improvement and Recovery^c^** | | | **Reliable Improvement or Recovery^d^** | | |
| **Engagement Group** | **NAT** | **AT** | **AB** | **NAT** | **AT** | **AB** | **NAT** | **AT** | **AB** | **NAT** | **AT** | **AB** | **NAT** | **AT** | **AB** |
| **Clinical Depression** |  |  |  |  |  |  |  |  |  |  |  |  |  |  |  |
| Short-term FUA | 618 | 1448 | 240 | 64.40% | 47.58% | 54.17% | 72.17% | 56.98% | 60.83% | 59.71% | 40.19% | 47.50% | 76.86% | 64.36% | 67.50% |
| 3-month FUA | 1610 | 2106 | 309 | 72.86% | 51.04% | 58.90% | 77.27% | 62.92% | 66.02% | 68.07% | 44.25% | 53.07% | 82.05% | 69.71% | 71.84% |
| 6-month FUA | 1035 | 1636 | 177 | 67.54% | 52.63% | 49.15% | 74.78% | 64.00% | 54.80% | 64.15% | 46.39% | 42.37% | 78.16% | 70.23% | 61.58% |
| 9-month FUA | 742 | 1252 | 101 | 64.42% | 51.68% | 52.48% | 72.51% | 66.29% | 62.38% | 60.38% | 46.41% | 48.51% | 76.55% | 71.57% | 66.34% |
| 12-month FUA | 310 | 402 | 27 | 65.16% | 56.72% | 66.67% | 72.58% | 66.42% | 66.67% | 63.23% | 51.00% | 62.96% | 74.52% | 72.14% | 70.37% |
| **Clinical Anxiety** |  |  |  |  |  |  |  |  |  |  |  |  |  |  |  |
| Short-term FUA | 858 | 2086 | 330 | 70.16% | 55.47% | 60.30% | 68.30% | 48.85% | 51.52% | 60.96% | 38.93% | 44.85% | 77.51% | 65.39% | 66.97% |
| 3-month FUA | 2282 | 3029 | 429 | 76.51% | 59.46% | 61.54% | 73.62% | 53.25% | 53.15% | 67.57% | 44.07% | 45.22% | 82.56% | 68.64% | 69.46% |
| 6-month FUA | 1466 | 2330 | 246 | 72.44% | 60.04% | 60.16% | 71.83% | 55.15% | 53.25% | 64.87% | 45.88% | 47.97% | 79.40% | 69.31% | 65.45% |
| 9-month FUA | 1028 | 1751 | 148 | 71.40% | 59.34% | 57.43% | 69.07% | 52.54% | 47.97% | 62.94% | 44.26% | 42.57% | 77.53% | 67.62% | 62.84% |
| 12-month FUA | 439 | 565 | 35 | 72.21% | 65.13% | 57.14% | 70.62% | 60.18% | 65.71% | 63.10% | 53.10% | 54.29% | 79.73% | 72.21% | 68.57% |
| **Clinical Depression or Anxiety** |  |  |  |  |  |  |  |  |  |  |  |  |  |  |  |
| Short-term FUA | 955 | 2300 | 365 | 73.72% | 59.43% | 64.66% | 76.48% | 61.88% | 65.63% | 67.12% | 46.00% | 53.15% | 82.20% | 71.52% | 73.15% |
| 3-month FUA | 2555 | 3372 | 483 | 78.71% | 62.93% | 66.46% | 80.95% | 66.14% | 68.38% | 71.90% | 50.65% | 53.42% | 85.71% | 75.09% | 76.60% |
| 6-month FUA | 1647 | 2608 | 283 | 75.17% | 63.31% | 59.72% | 77.79% | 67.69% | 63.41% | 69.58% | 52.80% | 51.24% | 82.94% | 75.08% | 69.26% |
| 9-month FUA | 1170 | 1962 | 167 | 73.42% | 62.23% | 60.48% | 76.08% | 67.48% | 63.75% | 66.92% | 51.43% | 51.50% | 81.20% | 74.72% | 68.86% |
| 12-month FUA | 494 | 634 | 39 | 75.30% | 67.35% | 58.97% | 76.63% | 71.46% | 66.27% | 69.03% | 58.68% | 56.41% | 81.98% | 77.60% | 69.23% |
| *Notes*. NAT: no additional therapy; AT: additional therapy; AB: additional booster; PHQ-9: 9-item Patient Health Questionnaire; GAD-7: 7-item Generalized Anxiety Disorder scale. AT and AB groups may or may not be currently enrolled in additional therapy or boosters during the FUA time periods. | | | | | | | | | | | | | | | |
| a Reliable Improvement: ≥ 4 decrease on the FUA GAD-7 among those with baseline GAD-7 ≥ 8 and/or ≥ 6 decrease on the FUA PHQ-9 among those with baseline PHQ-9 ≥ 10  b Recovery: FUA GAD-7 < 8 among those with baseline GAD-7 ≥ 8 and/or FUA PHQ-9 < 10 among those with baseline PHQ-9 ≥ 10  c Reliable Improvement and Recovery: ≥ 4 decrease on the FUA GAD-7 and FUA GAD-7 < 8 among those with baseline GAD-7 ≥ 8; and/or, ≥ 6 decrease on the FUA PHQ-9 and FUA PHQ-9 < 10 among those with baseline PHQ-9 ≥ 10  d Reliable Improvement or Recovery: ≥ 4 decrease on the FUA GAD-7 or FUA GAD-7 < 8 among those with baseline GAD-7 ≥ 8; and/or, ≥ 6 decrease on the FUA PHQ-9 or FUA PHQ-9 < 10 among those with baseline PHQ-9 ≥ 10 | | | | | | | | | | | | | | | |

Supplementary Table 5 includes the growth curve models depicting trajectories of PHQ-9 (Clinical Depression) and GAD-7 (Clinical Anxiety) symptoms on in-care assessments from the initial BCT treatment episode.

| **Supplementary Table 5. Growth Curve Model Results of In-care Assessments of Depression and Anxiety Symptoms** | | | | |
| --- | --- | --- | --- | --- |
|  | **Clinical Depression** | | **Clinical Anxiety** | |
|  | Model 1: Without post-care engagement groups | Model 2: Interaction with post-care engagement groups | Model 1: Without post-care engagement groups | Model 2: Interaction with post-care engagement groups |
| Intercept | 12.47 (12.41, 12.52) | 12.47 (12.40, 12.53) | 11.17 (11.12, 11.22) | 11.15 (11.10, 11.21) |
|  | t = 435.28*** | t = 375.45*** | t = 428.28*** | t = 382.92*** |
| Week | -1.23 (-1.25, -1.22) | -1.28 (-1.30, -1.26) | -1.03 (-1.04, -1.02) | -1.07 (-1.09, -1.06) |
|  | t = -147.03*** | t = -130.08*** | t = -167.57*** | t = -148.07*** |
| Week^2^ | 0.05 (0.05, 0.05) | 0.05 (0.05, 0.05) | 0.04 (0.04, 0.04) | 0.04 (0.04, 0.04) |
|  | t = 98.93*** | t = 86.41*** | t = 113.50*** | t = 99.25*** |
| Additional Therapy |  | 0.01 (-0.13, 0.15) |  | 0.08 (-0.02, 0.19) |
|  |  | t = 0.11 |  | t = 1.58 |
| Additional Booster |  | -0.05 (-0.33, 0.22) |  | 0.01 (-0.20, 0.21) |
|  |  | t = -0.37 |  | t = 0.06 |
| Week*Additional Therapy |  | 0.20 (0.16, 0.24) |  | 0.18 (0.15, 0.21) |
|  |  | t = 9.90*** |  | t = 12.36*** |
| Week*Additional Booster |  | 0.09 (0.01, 0.17) |  | 0.08 (0.02, 0.13) |
|  |  | t = 2.26* |  | t = 2.59** |
| Week^2^*Additional Therapy |  | -0.01 (-0.01, -0.01) |  | -0.01 (-0.01, -0.01) |
|  |  | t = -7.61*** |  | t = -10.19*** |
| Week^2^*Additional Booster |  | -0.004 (-0.01, 0.0004) |  | -0.005 (-0.01, -0.001) |
|  |  | t = -1.81 |  | t = -2.78** |
| Log Likelihood | -359,834.50 | -359,768.40 | -490,855.60 | -490,742.70 |
| Akaike Inf. Crit. | 719,689.10 | 719,568.90 | 981,733.20 | 981,519.30 |
| Bayesian Inf. Crit. | 719,786.70 | 719,725.20 | 981,844.40 | 981,691.20 |
| *Notes.* **p*<.05, ***p* <.01, *** *p* <.001  Clinical Depression: random client-level effects for the intercept, linear, and quadratic components; Clinical Anxiety: random provider-level effect for the intercept, random client-level effects for the intercept, linear, and quadratic components. | | | | |
|  | | | | |

Supplementary Table 6 includes sensitivity analyses around missingness on FUA. The robustness of the full-information maximum likelihood missing data handling used in the present study was reinforced in all analyses through the inclusion of client FUA eligibility group and the corresponding interactions in the primary analysis, as well as incorporation of the final in-care symptom assessment as the initial PHQ-9 or GAD-7 score, which ensures that this correlate of missingness is incorporated into both the primary and sensitivity analytic models. In addition, Models 1 and 2 in Supplementary Table 6 describe sensitivity analyses for the analytic models presented in Table 4 that incorporate clients who were excluded from the primary analysis for not providing an FUA (NPHQ-9 = 10,463; NGAD-7 = 14,635). For Model 1 using both PHQ-9 and GAD-7, the pattern of coefficients for the linear (Week) and quadratic (Week2) coefficients maintained the same sign across the primary (Table 4) and sensitivity (Supplementary Table 6) analyses, and although the coefficients were all statistically significant, the linear and quadratic effects were weaker (closer to 0) in the sensitivity analysis.

A slightly different pattern of findings emerged for the sensitivity analysis featuring the Model 2 specification provided in Supplementary Table 6, relative to Model 2 presented in the primary analysis. Specifically, a negative linear (Week) coefficient emerged that was qualified by a positive quadratic (Week2) component, suggesting a small initial decrease in depression and anxiety symptoms that became more positive over time, among clients who did not return to care. However, the interaction terms involving the linear and quadratic time effects, and the return-to-care group indicators maintained the same sign as the primary analysis, though the interactions involving the Week term were slightly larger than the primary analysis. Overall, the pattern of findings was largely consistent with the primary analysis presented in Table 4. Specifically, clients receiving an Additional Booster or Additional Therapy exhibited an initial increase in depression and anxiety symptoms that became flatter (more negative) over the follow-up period.

A final sensitivity analysis incorporating known predictors of missingness, including client’s demographic characteristics, baseline PHQ-9 and GAD-7 scores from their active treatment episode, the corresponding final in-care GAD-7 or PHQ-9 score, as well as the number of sessions attended and treatment duration were included in the Model 3 specification presented in Supplementary Table 6. Compared to the Model 2 specification for the Clinical Anxiety analysis, the first-order linear and quadratic coefficients were both weaker (closer to 0) indicating that the trajectories for clients who did not return to care were essentially linear and flat. A slightly different pattern emerged for the Clinical Depression group, which was characterized by positive first-order linear and quadratic coefficients that were also very close to zero, which are generally consistent with a flat post-care trajectory. In addition, the coefficients describing the return to care group interaction terms were consistent with a flatter post-treatment symptom trajectory for both groups, and smaller differences across groups of clients who returned to care and those who did not. Overall, these sensitivity analyses suggest that including cases who did not provide any FUAs in order to strictly satisfy the missing data assumptions has no meaningful impact on the primary study conclusions.

| Supplementary Table 6. Growth Curve Model Results of Final In-care and Follow-up Assessments of Depression and Anxiety Symptoms, Including Participants who Engaged in Care with and without FUA | | | | | | | | | | | | |
| --- | --- | --- | --- | --- | --- | --- | --- | --- | --- | --- | --- | --- |
| Clinical Depression | | | | | | Clinical Anxiety | | | | | | |
|  |  | Model 1: Without post-care engagement groups | Model 2: Interaction with post-care engagement groups | Model 3: Includes demographic variables | |  |  | Model 1: Without post-care engagement groups | | Model 2: Interaction with post-care engagement groups | | Model 3: Includes demographic variables |
| Intercept | | 6.82 (6.75, 6.90) | 6.29 (6.20, 6.37) | | 6.59 (6.50, 6.68) | Intercept | | 6.29 (6.21, 6.36) | 5.77 (5.69, 5.85) | | 6.02 (5.95, 6.09) | |
|  | | t = 180.37*** | t = 141.85*** | | t = 139.95*** |  | | t = 167.14*** | t = 143.95*** | | t = 168.56*** | |
| Week | | 0.08 (0.07, 0.09) | -0.08 (-0.09, -0.06) | | 0.003 (-0.01, 0.01) | Week | | 0.08 (0.07, 0.09) | -0.06 (-0.07, -0.05) | | -0.01 (-0.02, -0.004) | |
|  | | t = 19.99*** | t = -11.29*** | | t = 0.51 |  | | t = 25.63*** | t = -11.62*** | | t = -2.97** | |
| Week^2^ | | -0.001 (-0.002, -0.001) | 0.003 (0.002, 0.003) | | 0.001 (0.001, 0.001) | Week^2^ | | -0.001 (-0.001, -0.001) | 0.002 (0.002, 0.002) | | 0.001 (0.001, 0.001) | |
|  | | t = -13.20*** | t = 14.66*** | | t = 6.53*** |  | | t = -17.01*** | t = 15.82*** | | t = 10.60*** | |
| Additional Therapy | |  | 1.30 (1.13, 1.48) | | 0.85 (0.73, 0.97) | Additional therapy | |  | 1.29 (1.16, 1.41) | | 0.90 (0.81, 0.99) | |
|  | |  | t = 14.64*** | | t = 13.43*** |  | |  | t = 20.09*** | | t = 19.20*** | |
| Additional Booster | |  | 0.44 (0.09, 0.79) | | 0.46 (0.21, 0.71) | Booster | |  | 0.50 (0.24, 0.75) | | 0.48 (0.29, 0.66) | |
|  | |  | t = 2.48* | | t = 3.63*** |  | |  | t = 3.83*** | | t = 5.02*** | |
| Final In-care GAD-7 | |  |  | | 0.74 (0.73, 0.75) | Final In-care PHQ-9 | |  |  | | 0.60 (0.59, 0.61) | |
|  | |  |  | | t = 120.77*** |  | |  |  | | t = 135.28*** | |
| Age | |  |  | | -0.01 (-0.01, -0.003) | Age | |  |  | | -0.003 (-0.01, 0.001) | |
|  | |  |  | | t = -3.11** |  | |  |  | | t = -1.46 | |
| Gender | |  |  | |  | Gender | |  |  | |  | |
|  | *Male* |  |  | | -0.12 (-0.23, -0.01) |  | *Male* |  | |  | | -0.25 (-0.33, -0.17) |
|  |  |  |  | | t = -2.21* |  |  |  | |  | | t = -5.94*** |
|  | *Other/unknown gender* |  |  | | 0.75 (0.36, 1.14) |  | *Other/unknown gender* |  | |  | | -0.10 (-0.42, 0.21) |
|  |  |  |  | | t = 3.80*** |  |  |  | |  | | t = -0.65 |
| Race and Ethnicity | |  |  | |  | Race and Ethnicity | |  |  | |  | |
|  | *Asian or Pacific Islander* |  |  | | -0.20 (-0.34, -0.06) |  | *Asian or Pacific Islander* |  | |  | | 0.07 (-0.04, 0.17) |
|  |  |  |  | | t = -2.81** |  |  |  | |  | | t = 1.26 |
|  | *Black or African American* |  |  | | -0.16 (-0.35, 0.03) |  | *Black or African American* |  | |  | | -0.07 (-0.21, 0.08) |
|  |  |  |  | | t = -1.63 |  |  |  | |  | | t = -0.86 |
|  | *Hispanic or Latino* |  |  | | -0.21 (-0.38, -0.04) |  | *Hispanic or Latino* |  | |  | | -0.002 (-0.13, 0.13) |
|  |  |  |  | | t = -2.46* |  |  |  | |  | | t = -0.03 |
|  | *Multiple* |  |  | | -0.02 (-0.21, 0.17) |  | *Multiple* |  | |  | | -0.04 (-0.18, 0.10) |
|  |  |  |  | | t = -0.20 |  |  |  | |  | | t = -0.56 |
|  | *Other* |  |  | | -0.08 (-0.45, 0.29) |  | *Other* |  | |  | | 0.09 (-0.18, 0.35) |
|  |  |  |  | | t = -0.43 |  |  |  | |  | | t = 0.62 |
|  | *Prefer not to disclose/missing* |  |  | | 0.19 (-0.18, 0.55) |  | *Prefer not to disclose/missing* |  | |  | | 0.50 (0.23, 0.77) |
|  | |  |  | | t = 1.01 |  | |  |  | | t = 3.59*** | |
| Baseline PHQ-9 | |  |  | | 0.27 (0.25, 0.28) | Baseline PHQ-9 | |  |  | | -0.10 (-0.11, -0.09) | |
|  | |  |  | | t = 35.66*** |  | |  |  | | t = -23.74*** | |
| Baseline GAD-7 | |  |  | | -0.13 (-0.14, -0.12) | Baseline GAD-7 | |  |  | | 0.21 (0.20, 0.23) | |
|  | |  |  | | t = -20.74*** |  | |  |  | | t = 36.26*** | |
| # Therapy sessions completed | |  |  | | -0.04 (-0.07, -0.01) | # Therapy sessions completed | |  |  | | -0.06 (-0.08, -0.04) | |
|  | |  |  | | t = -2.82** |  | |  |  | | t = -5.63*** | |
| Duration of care (weeks) | |  |  | | -0.01 (-0.02, 0.01) | Duration of care (weeks) | |  |  | | 0.01 (-0.01, 0.02) | |
|  | |  |  | | t = -1.11 |  | |  |  | | t = 0.88 | |
| Week*Additional Therapy | |  | 0.22 (0.20, 0.23) | | 0.15 (0.13, 0.17) | Week*Additional therapy | |  | 0.18 (0.17, 0.20) | | 0.15 (0.13, 0.16) | |
|  | |  | t = 24.37*** | | t = 18.69*** |  | |  | t = 27.71*** | | t = 24.35*** | |
| Week*Additional Booster | |  | 0.22 (0.18, 0.25) | | 0.16 (0.13, 0.20) | Week*Additional Booster | |  | 0.21 (0.18, 0.24) | | 0.16 (0.14, 0.19) | |
|  | |  | t = 11.56*** | | t = 9.34*** |  | |  | t = 14.96*** | | t = 12.82*** | |
| Week^2^*Additional Therapy | |  | -0.01 (-0.01, -0.01) | | -0.004 (-0.005, -0.004) | Week^2^*Additional therapy | |  | -0.005 (-0.005, -0.004) | | -0.004 (-0.004, -0.004) | |
|  | |  | t = -25.26*** | | t = -21.40*** |  | |  | t = -28.44*** | | t = -26.90*** | |
| Week^2^*Additional Booster | |  | -0.004 (-0.01, -0.003) | | -0.003 (-0.004, -0.002) | Week^2^*Additional Booster | |  | -0.004 (-0.005, -0.003) | | -0.003 (-0.004, -0.002) | |
|  | |  | t = -7.94*** | | t = -6.16*** |  | |  | t = -10.56*** | | t = -8.32*** | |
| Log Likelihood | | -132,484.00 | -131,974.10 | | -125,414.30 | Log Likelihood | | -177,940.70 | -177,185.90 | | -168,648.30 | |
| Akaike Inf. Crit. | | 264,982.00 | 263,974.30 | | 250,882.60 | Akaike Inf. Crit. | | 355,901.30 | 354,403.80 | | 337,356.70 | |
| Bayesian Inf. Crit. | | 265,042.90 | 264,087.40 | | 251,117.50 | Bayesian Inf. Crit. | | 355,991.70 | 354,548.30 | | 337,627.60 | |

*Notes.* **p*<.05, ***p* <.01, *** *p* <.001

Clinical Depression: random client-level effects for the intercept and linear components; Clinical Anxiety: random provider-level effect for the intercept and linear components, random client-level effects for the intercept and linear components; Centering: Age, Baseline PHQ-9/GAD-7, Final In-Care PHQ-9/GAD-7 are centered to the grand mean; # Therapy sessions completed and Duration of care (weeks) are centered to the grand median.

**Supplementary Figure 1:** Growth curve models of PHQ-9 follow-up assessments with the post-care engagement group interaction


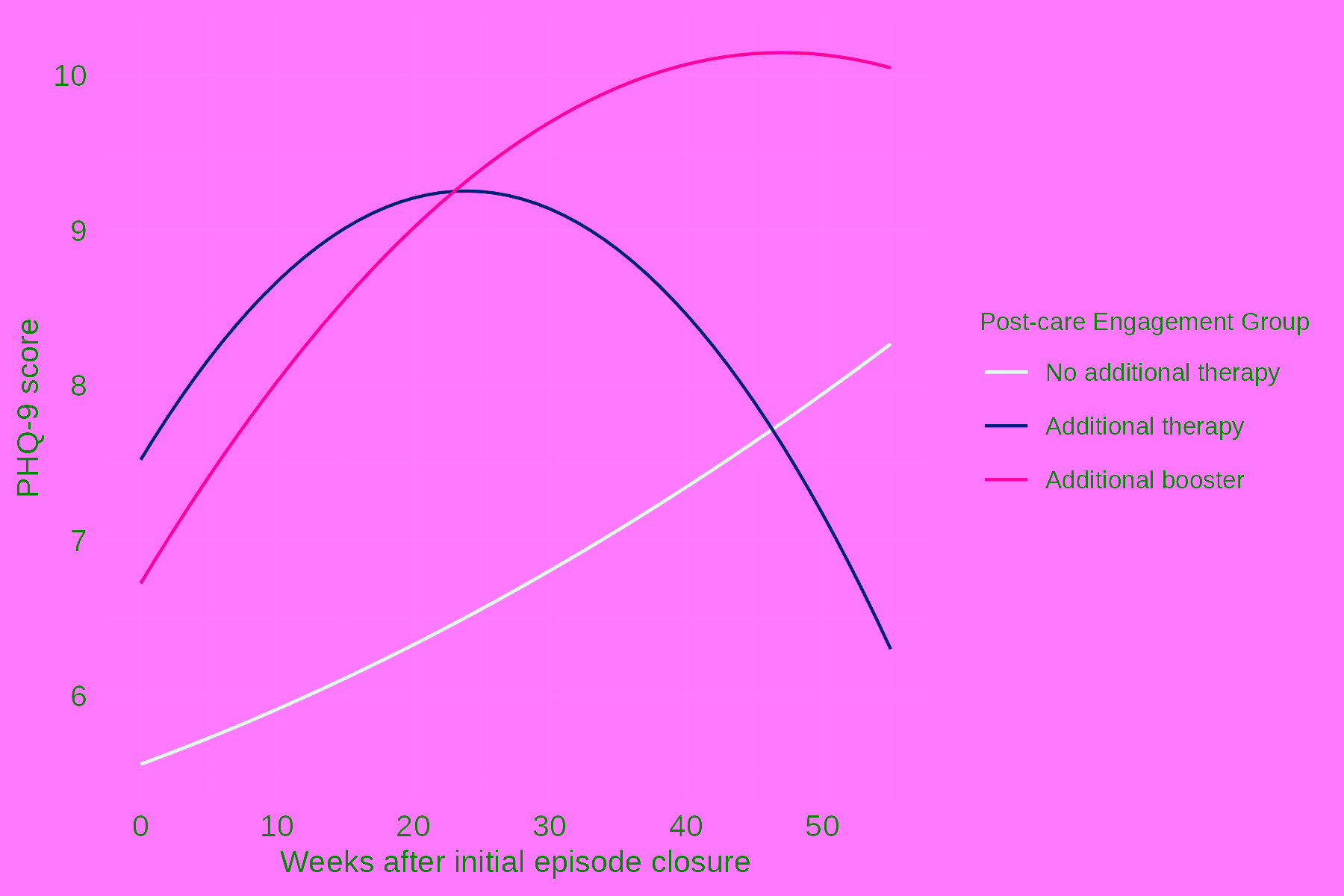


*Note*. Symptoms on the PHQ-9 are considered clinically elevated when they are greater than or equal to 10.

**Supplementary Figure 2:** Growth curve models of GAD-7 follow-up assessments with the post-care engagement group interaction


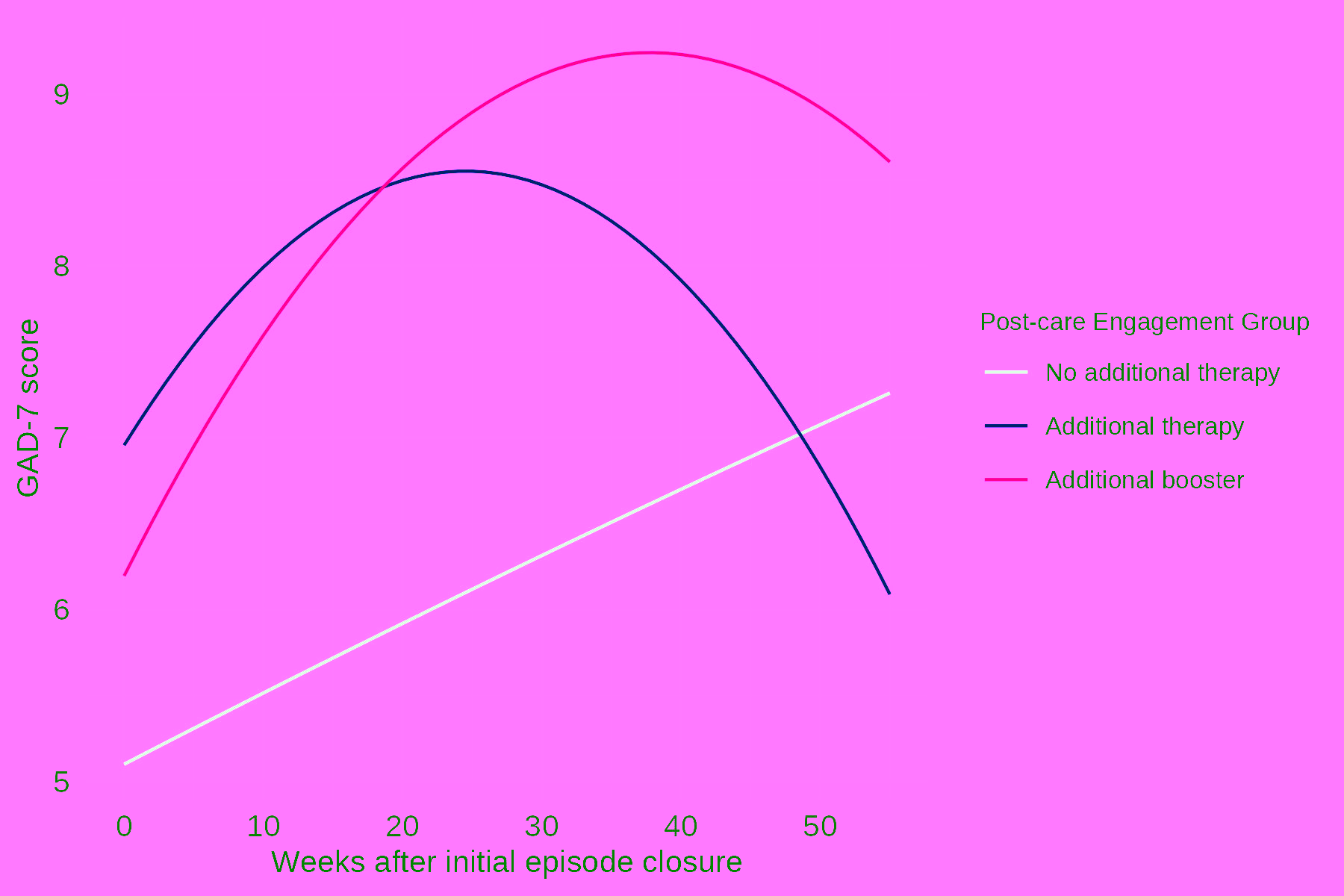


*Note*. Symptoms on the GAD-7 are considered clinically elevated when they are greater than or equal to 8.

**References**

Bates, D., Mächler, M., Bolker, B., Walker, S., 2015. Fitting linear mixed-effects models using lme4. J. Stat. Softw. 67, 1–48. https://doi.org/10.18637/jss.v067.i01

Lungu, A., Jun, J.J., Azarmanesh, O., Leykin, Y., Chen, C.E.-J., 2020. Blended care-cognitive behavioral therapy for depression and anxiety in real-world settings: pragmatic retrospective study. J. Med. Internet Res. 22, e18723. https://doi.org/10.2196/18723

Owusu, J.T., Wang, P., Wickham, R.E., Varra, A.A., Chen, C., Lungu, A., 2022. Real-world evaluation of a large-scale blended care-cognitive behavioral therapy program for symptoms of anxiety and depression. Telemed. E-Health. https://doi.org/10.1089/tmj.2021.0590

R Core Team, 2023. R: A language and environment for statistical computing. R Foundation for Statistical Computing, Vienna, Austria.
